# Phylodynamics and Codon Usage Pattern Analysis of Broad Bean Wilt Virus 2

**DOI:** 10.3390/v13020198

**Published:** 2021-01-28

**Authors:** Zhen He, Zhuozhuo Dong, Lang Qin, Haifeng Gan

**Affiliations:** 1 School of Horticulture and Plant Protection, Yangzhou University, Yangzhou 225009, China; 18852727287@163.com (Z.D.); 18252788281@163.com (L.Q.); ghf18362828428@163.com (H.G.); 2 Joint International Research Laboratory of Agriculture and Agri-Product Safety of Ministry of Education of China, Yangzhou University, Yangzhou 225009, China

**Keywords:** broad bean wilt virus 2, codon usage pattern, natural selection, host adaptation

## Abstract

Broad bean wilt virus 2 (BBWV-2), which belongs to the genus *Fabavirus* of the family *Secoviridae*, is an important pathogen that causes damage to broad bean, pepper, yam, spinach and other economically important ornamental and horticultural crops worldwide. Previously, only limited reports have shown the genetic variation of BBWV2. Meanwhile, the detailed evolutionary changes, synonymous codon usage bias and host adaptation of this virus are largely unclear. Here, we performed comprehensive analyses of the phylodynamics, reassortment, composition bias and codon usage pattern of BBWV2 using forty-two complete genome sequences of BBWV-2 isolates together with two other full-length RNA1 sequences and six full-length RNA2 sequences. Both recombination and reassortment had a significant influence on the genomic evolution of BBWV2. Through phylogenetic analysis we detected three and four lineages based on the ORF1 and ORF2 nonrecombinant sequences, respectively. The evolutionary rates of the two BBWV2 ORF coding sequences were 8.895 × 10^−4^ and 4.560 × 10^−4^ subs/site/year, respectively. We found a relatively conserved and stable genomic composition with a lower codon usage choice in the two BBWV2 protein coding sequences. ENC-plot and neutrality plot analyses showed that natural selection is the key factor shaping the codon usage pattern of BBWV2. Strong correlations between BBWV2 and broad bean and pepper were observed from similarity index (SiD), codon adaptation index (CAI) and relative codon deoptimization index (RCDI) analyses. Our study is the first to evaluate the phylodynamics, codon usage patterns and adaptive evolution of a fabavirus, and our results may be useful for the understanding of the origin of this virus.

## 1. Introduction

Broad bean wilt virus 2 (BBWV-2) belongs to the genus *Fabavirus* of the *Comovirinae* subfamily, *Secoviridae* family. BBWV-2 is an important pathogen causing extensive damage in broad bean, pepper, yam, spinach and other economically important horticultural and ornamental crops worldwide [1,2,3,4,5,6], and it is transmitted by aphids in a nonpersistent manner. The virions of BBWV-2 contain two proteins (large and small coat proteins) which form an icosahedral particle. BBWV-2 comprises bipartite positive-sense single-stranded RNA molecules with a genome size of approximately 6 and 4 kb. RNA1 encodes a single large polyprotein with functional proteins involved in genome replication and expression that are produced by proteolytic cleavage. Similarly, RNA2 also encodes a single large polyprotein, which is proteolytically processed into three functional proteins, including a movement protein and two coat proteins.

Sixty-one triplet codons encode all 20 amino acids, and thus several codons encode the same amino acid. This phenomenon is termed synonymous codons [7,8]. Generally, the unequal preference for specific codons over other synonymous codons by various organisms or even in different gene groups of the same genome creates a bias in codon usage, and this phenomenon is known as codon usage bias (CUB) [9,10,11,12]. Currently, several factors have been reported to drive codon usage patterns, such as compositional constraints, hydrophobicity, mutation pressure, gene length, replication, natural selection, selective transcription, secondary protein structure, gene function, and the external environment [7,9,10,12,13,14,15,16,17]. Mainly, two models including mutational/neutral and natural/translational selection, may explain the codon usage bias [8,9,10]. CUB in viruses is expected to affect their survival, fitness, evolution, adaption, and avoidance of host cell responses [7,13]. Until now, only several reports have described the influence of codon usage in the evolution of plant viruses, such as begomoviruses [18], citrus tristeza virus (CTV) [19], rice black-streaked dwarf virus (RBSDV) [20], rice strape virus (RSV) [21], papaya ringspot virus (PRSV) [22], potato virus M (PVM) [23], and sugarcane mosaic virus (SCMV) [24].

The genetic variation of BBWV-2 has been described based on analyses of partial or complete genome sequences [2,25]. To date, 42 complete genome sequences of BBWV-2 isolates from China, Japan, Philippines, Singapore, and South Korea, together with two full-length RNA1 sequences and five full-length RNA2 sequences, have been reported [4,25,26,27,28,29,30,31]. However, these studies did not clearly report on the synonymous codon usage pattern of BBWV-2.

In this study, we performed a detailed analysis of the traditional phylogeny, reassortment and codon usage of BBWV-2 based on 44 full-length RNA1 and 47 full-length RNA2 sequences. The analysis explores the factors shaping the codon usage patterns of BBWV-2 and provides novel insight into the genetic divergence of BBWV-2. To the best of our knowledge, this study is the first to evaluate the codon usage patterns of a fabavirus.

## 2. Materials and Methods

### 2.1. Virus Isolates

Forty-four full-length RNA1 and 47 full-length RNA2 sequences of BBWV-2 were retrieved from GenBank. The details of those sequences, such as host origins, collection time, locations and geographical, are shown in Supplementary Materials Table S1.

### 2.2. Recombination Analysis

Forty-four full-length RNA1 and 47 full-length RNA2 sequences of BBWV-2 were aligned using CLUSTAL X2 [32]. TRANSALIGN software (supplied kindly by Prof. Georg Weiller, Australian National University, Canberra, Australia) was used to support a degapped alignment of the encoded amino acids. Putative recombination events of the aligned BBWV-2 sequences were identified by BOOTSCAN, CHIMAERA, GENECONV, MAXCHI, RDP, SISCAN and 3SEQ programs [33,34,35,36,37,38,39] in the RDP4 software package [40]. The phylogenetic approach was used to verify the parent/donor assignments in the RDP4 package. And these analyses were calculated by different detection programs with default settings. The putative recombinants were supported by at least three different methods in the RDP4 package with an associated *p*-value of <1.0 × 10^−6^.

### 2.3. Phylogenetic and Evolution Dynamic Analysis

The phylogenetic relationships of the two ORF sequences of BBWV-2 were determined using the maximum-likelihood (ML) method in PhyML v3.0 [41] and the neighbour-joining (NJ) method implemented in MEGA vX [42]. The best-fitting models of the two datasets for the ML tree were selected by jModeltest v0.1.1 [43] according to the Akaike Information Criterion score. GTR with a proportion of invariable sites and a gamma distribution (GTR+I+г4) provided the best fit for both ORF coding sequences. For the ML analysis, branch support was calculated by a bootstrap analysis based on 1000 pseudoreplicates; meanwhile, for the NJ analysis, Kimura’s two-parameter [44] option was used to evaluate 1000 bootstrap replications. The lineages were defined based on bootstrap values. When the node with high bootstrap values on both ML and NJ tree, we consider it as a defined lineage. The ML or NJ trees were displayed with TreeView [45]. Generally, two segments of the same BBWV-2 isolate should be divided into same lineage based on ORF1 and ORF2 trees. We considered that reassortment occurred when the two segments of BBWV-2 isolate divided into different lineages based on ORF1 and ORF2 trees. The pairwise nucleotide sequence identity scores were represented as a distribution plot using SDT version 1.2 software (available from http://web.cbio.uct.ac.za/SDT) [46].

All nonrecombinant sequences obtained were used to estimate the evolutionary rate and timescale by BEAST v1.10.4 software [47]. Bayes factors were used to select the best-fitting molecular-clock models. The strict molecular clocks, uncorrelated lognormal, and uncorrelated exponential models [48] were also compared with the exponential growth, logistic growth, constant population size, Bayesian skyline plot, and expansion growth demographic models. A total of 6 × 10^8^-step MCMC chains were explored every 10^4^ steps, and the first 10% of samples were removed as burn-in. Tracer v1.7 [49] was used to check the estimation of the relevant evolutionary parameters. To check the temporal signal, ten data-randomized replicates of the data were produced. The mean estimate from the original data out of 95% CIs of the date-randomized replicates is considered the criterion for clear temporal structure [50,51].

### 2.4. Nucleotide Composition Analysis

In total, five nonbiased codons, including three termination codons (UAA, UGA, and UAG), AUG (encoding only Met), and UGG (encoding only Trp), were removed, and the component parameters of both BBWV2 ORF sequences were calculated. The total content of AU and GC and the entire nucleotide composition (A, U, G and C %) of the two ORF data sets were calculated by BioEdit [52]. The nucleotide composition at the third codon position of the two BBWV-2 ORF sequences (A3, U3, G3 and C3%) were determined using the CodonW 1.4.2 package. EMBOSS explorer (http://www.bioinformatics.nl/emboss-explorer/) was used to calculate the GC content at the 1st, 2nd and 3rd codon positions (GC1, GC2, GC3) and GC12 (the mean of GC1 and GC2).

### 2.5. Effective Number of Codon (ENC) Analysis

ENC values ranging from 20 (only one synonymous codon is used, an extreme codon usage bias) to 61 (the synonymous codons are used equally, no bias), indicating the degree of codon usage bias [53], were calculated by CodonW v1.4.2 software. ENC values were estimated as follows:

(1) $$\mathrm{ENC}=2+\frac{9}{{\bar{F}}_{2}}+\frac{1}{{\bar{F}}_{3}}+\frac{5}{{\bar{F}}_{4}}$$

where ${\bar{F}}_{k}$ (*k* = 2, 3, 4, 6) is the average values for *F_k_*, while *k* is the k-fold degenerate amino acids. Here, *F_k_* is calculated as follows:

(2) $$F_{k}=\frac{nS-1}{n-1}$$

where *n* is the total occurrence number of the codon for the corresponding amino acid; meanwhile,

(3) $$S=\overset{k}{\underset{i=1}{\sum }}\;{\left(\frac{n_{i}}{n}\right)}^{2}$$

where *n_i_* represents the total number of the i-th codon for that amino acid.

Here, the ENC was assessed to compute the absolute codon usage bias of both BBWV-2 ORF sequences regardless of the number of amino acids and the gene lengths. Generally, ENC values ≤ 35 indicate strong codon bias. It is accepted that the smaller the ENC value, the stronger the codon preference.

### 2.6. ENC-Plot Analysis

To investigate the role of mutation pressure in codon usage bias, a ENC-plot (ENC value in the ordinate against GC3s value in the abscissa) analysis was used. When the codon usage bias is only determined by the mutation pressure factor, the points will lie on or around the standard curve. Otherwise, other factors also contribute, for example, natural selection. The expected ENC was calculated using the following formula:

(4) $$\mathrm{ENC}\;\mathrm{expected}=2+s+\left(\frac{29}{s^{2}+\left(1-s\right)2}\right)$$

where s means the composition of GC3s.

### 2.7. Relative Synonymous Codon Usage (RSCU) Analysis

The ratio between the observed usage frequency and the expected usage frequency is termed as the RSCU value of a codon [54]. RSCU values were calculated as follows:

(5) $${\mathrm{RSCU}}_{\mathrm{ij}}=\frac{g_{\mathrm{ij}}}{\sum_{j}^{\mathrm{ni}}\;g_{\mathrm{ij}}}\times \mathrm{ni}$$

where RSCU_ij_ represents the value of the *i*-th codon for the *j*-th amino acid, and g_ij_ means the observed number of *i*-th codons for the *j*-th amino acid which has “n_i_” kinds of synonymous codons. And the RSCU values of 1 indicate no bias for the codon, whereas codons with RSCU values more than 1.6 and smaller than 0.6 are considered to be “over-represented” and “under-represented”, respectively. The RSCU values of the two BBWV2 ORF sequences were calculated using MEGA X software.

### 2.8. Principal Component (PCA) Analysis

A multivariate statistical method (principal component analysis) was used to identify the correlations between samples and variables. After removing the codons UAA, UAG, UGA, UGG, and AUG, each strain of two ORF data sets was represented as a 59-dimensional vector, where each dimension corresponds to each sense codon’s RSCU value [21,55]. A PCA analysis was performed using Origin 8.0 (OriginLab, Northampton, MA, USA).

### 2.9. Parity Rule 2 Analysis (PR2)

A PR2 plot analysis was performed to calculate the effects of mutation and natural selection on the codon usage of the two BBWV2 ORF sequences. The PR2 plot graphs A3/(A3 + U3) in the ordinate against G3/(G3 + C3) in the abscissa [21,55]. The centre of the plot (the slope is 0.5) indicates no bias between natural selection and mutation pressure.

### 2.10. Neutrality Analysis

The influence of mutation and natural selection bias on codon usage were analysed using a neutrality plot. Neutrality plot graphs GC3 in the abscissa and GC12 in the ordinate. The mutational force was indicated using the slope of the regression line which plotted between the GC12 and GC3 contents [21,55]. A slope of the regression lines on or around 1.0 indicates no or weak selection pressure. However, the codon usage bias was clearly influenced by natural selection when the regression curves deviated from the diagonal line.

### 2.11. Codon Adaptation Index (CAI) Analysis

The CAI value, which ranged from 0 to 1, is calculated by the CAIcal SERVER (http://genomes.urv.cat/CAIcal/RCDI/). It is used to predict the adaptation of the two ORFs of BBWV2 to their host. All above BBWV2 isolates were compared to each host. In general, the higher the CAIs, the stronger the adaptability to the host.

### 2.12. Relative Codon Deoptimization Index (RCDI) Analysis

To determine the trends of the codon deoptimization, RCDI values for the two BBWV2 ORF sequences were computed by the RCDI/eRCDI server (http://genomes.urv.cat/CAIcal/RCDI/). RCDI values equal to 1 indicate that the virus has a host-adapted codon usage pattern. In contrast, RCDI values> 1 indicate less adaptability.

### 2.13. Similarity Index (SiD) Analysis

The SiD analysis was employed to evaluate the influence of the codon usage bias of hosts on the two BBWV2 ORFs. The SiD values was estimated as follows:

(6) $$R\left(A,B\right)=\frac{\sum_{i=1}^{59}\;a_{i}\;b_{i}}{\sqrt{\sum_{i=1}^{59}\;b_{i}\;{}^{2}\sum_{i=1}^{59}\;a_{i}\;{}^{2}}}$$

(7) $$D\left(A,B\right)=\frac{1-R\left(A,B\right)}{2}$$

where *a_i_* is the RSCU values of 59 synonymous codons of the BBWV2 coding sequences, and *b_i_* represents the identical codons’ RSCU values of the host. SiD [D(A, B)] value, which ranged from 0 to 1.0, represents the potential effect of the entire codon usage of hosts on the BBWV-2 genes. Normally, higher SiD values indicate that the viruses’ host plays a significant role in its codon usage.

### 2.14. Gravy and Aroma Statistics

A Gravy value ranging from −2 to 2 indicates the effect of protein hydrophobicity on codon usage bias. It is determined by CodonW (v1.4.2). Meanwhile, aroma value represents the influence of aromatic hydrocarbon proteins on codon usage bias.

### 2.15. Statistical Analysis

The relationships between the GC3s, GC ENC, Aroma, and Gravy and the first two principal component axes were measured using a Spearman’s rank correlation analysis. A *p* value < 0.01 (**) shows an extremely significant relationship while 0.01 < *p* < 0.05 (*) represents a significant relationship. All of the above statistical analyses were estimated by Origin 8.0.

## 3. Results

### 3.1. Recombination and Phylogenetic Analysis

Recombination can influence the topology of a phylogenetic tree and overall codon usage patterns at either the genome or gene level [56,57]. Thus, we first detected the presence of potential recombinants in the 44 full-length RNA1 and 47 full-length RNA2 sequences. Two and six clear recombinants from 44 full-length RNA1 and 47 full-length RNA2 sequences were observed (Table 1), respectively, and these recombinants were excluded from further analysis. The nonrecombinant BBWV-2 coding sequences mainly isolated from broad bean (RNA1 *n* = 6, RNA2 *n* = 5), pepper (RNA1 *n* = 18, RNA2 *n* = 18), spinach (RNA1 *n* = 3, RNA2 *n* = 3), and yam (RNA1 *n* = 4, RNA2 *n* = 4) were used in the following phylogenetic and codon usage analyses.

**Figure 1:**
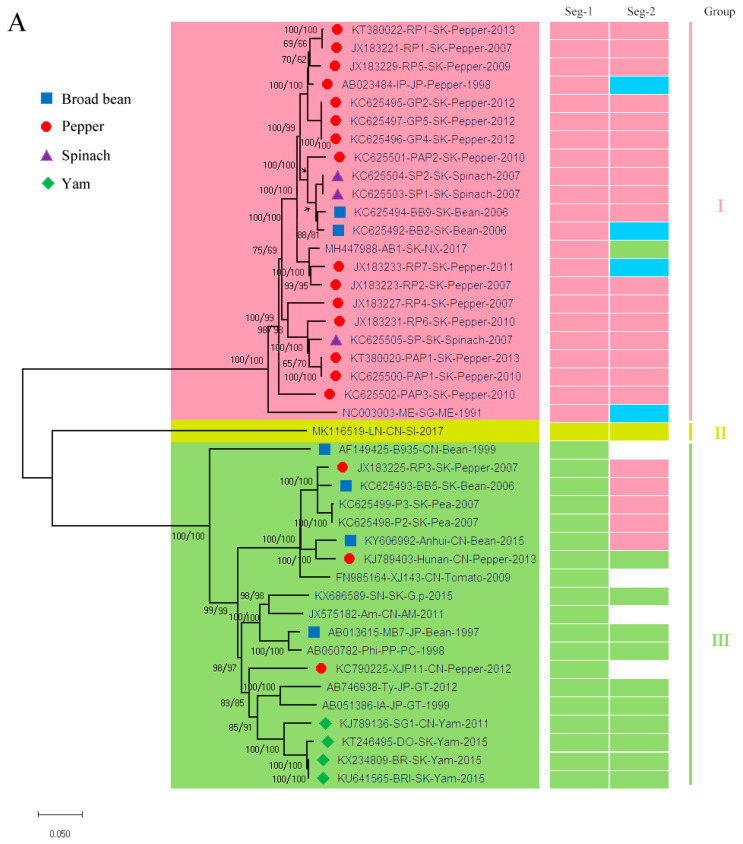

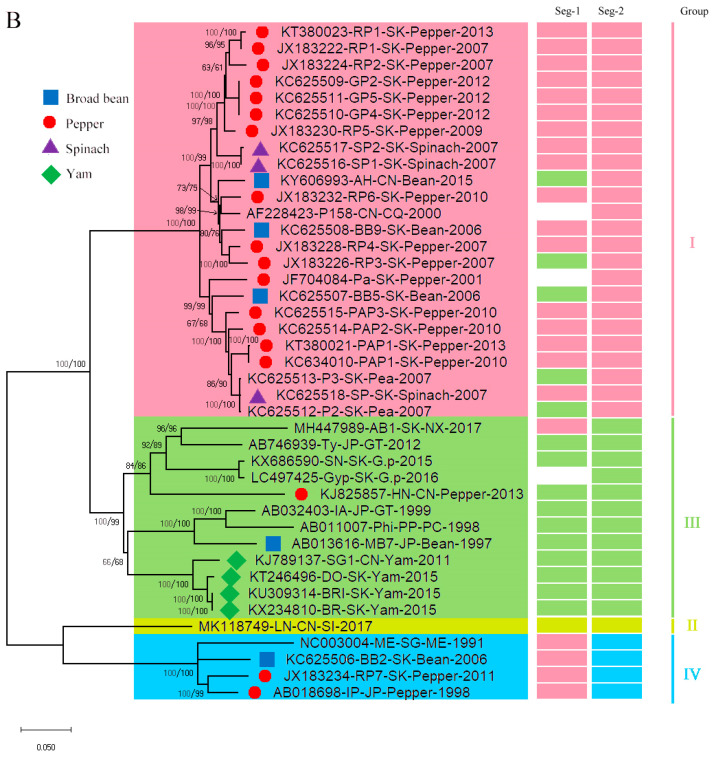
The maximum-likelihood (ML) trees calculated from the ORF1 (**A**) and ORF2 (**B**) sequences of nonrecombinant broad bean wilt virus 2. Numbers at each node indicate the percentage of bootstrap samples in the NJ and ML trees. The horizontal branch length is drawn to scale with the bar indicating 0.05 nt replacements per site.

**Figure 2:**
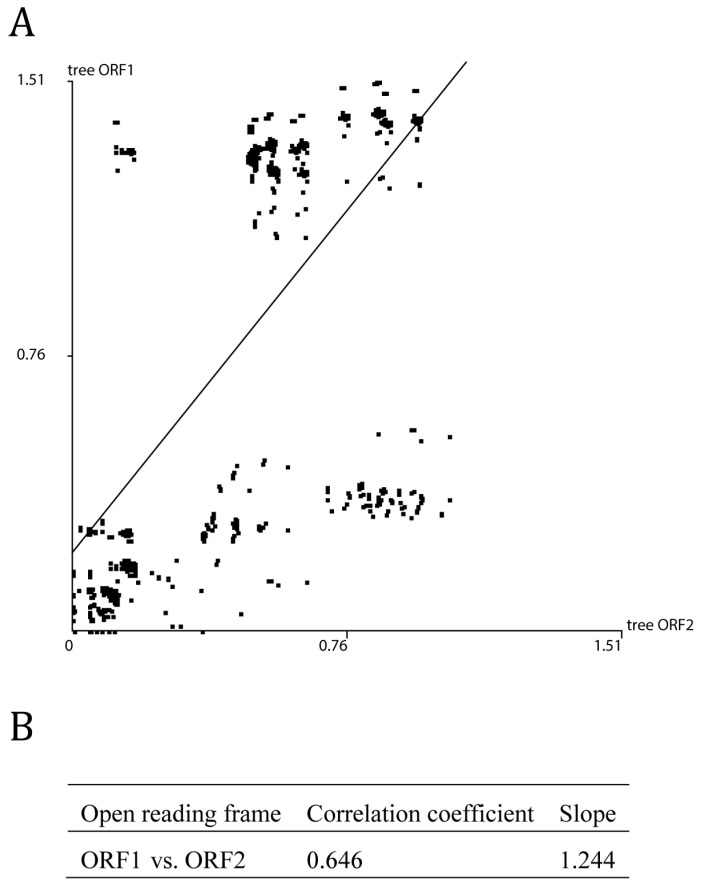
Graphs comparing patristic distances in pairs of maximum-likelihood trees based on the ORF1 and ORF2 sequences of nonrecombinant broad bean wilt virus 2. (**A**) Graphs comparing patristic distances; (**B**) correlation coefficient of ORF1 and ORF2.

The phylogenetic analyses were conducted using ML methods based on the two ORFs’ nonrecombinant sequences (Figure 1), respectively. Three and four lineages were formed based on the ORF1 and ORF2 coding sequences, respectively (Figure 1). Four isolates in lineage I of the ORF1 ML tree were clustered into lineage IV in the ML tree of ORF2 (Figure 1). These lineages did not reflect clear host and geographical origins. The ML trees of the ORF 1 and 2 coding sequences were compared using PATRISTIC software. The distance plots of the ORF1 distances against the ML trees of the ORF2 genes showed distinct lineages (Figure 2). Similar, our time-scaled maximum clade credibility (MCC) tree also indicated three and four lineages based on the ORF1 and ORF2 coding sequences, respectively (Supplementary Materials Figure S1). The pairwise identity of ORF1 and ORF2 were approximately 77.59–100% and 78.17–100%, respectively.

### 3.2. Reassortment Analysis

Generally, reassortment can influence the rapid genomic and phenotypic changes for viruses with segmented genomes by coinfecting different viral strains exchanges entire segments. Our ML and time-scaled phylogenies distinguished three phylogenetic groups for Segment 1 (ORF1) and four groups for Segment 2 (ORF2) with high bootstrap or posterior support (Figure 1 and Supplementary Materials Figure S1). Ten isolates appear to be reassorted (23.8%) (Figure 1A) among the 42 full-length BBWV-2 isolates. For example, the AB1 isolate (MH447988) from South Korea was clustered into lineage I in the ORF1 ML tree, whereas it was divided into lineage III in the ORF2 ML tree (Figure 1A). All four isolates in lineage IV in the ORF2 ML tree were clustered into Lineage I in the ORF1 ML tree (Figure 1A). In addition, five isolates (the RP3, BB5, P3, and P2 isolates from South Korea and the Anhui isolate from China) in lineage III for the segment 1 tree were clustered into lineage I for the segment 2 tree (Figure 1A). Furthermore, we performed reassortment analysis by RDP software using 38 BBWV2 artificially concatenated nonrecombinant sequences. This results also supported that ten isolates (IP, BB2, AB1, RP7, RP3, ME, BB5, P3, P2, and AH) appear to be reassorted (Supplementary Materials Table S2).

### 3.3. Evolutionary Dynamic Analysis

A Bayesian phylogenetic method in BEAST v1.10.4 [47] was used here to estimate the evolutionary rates and node ages of BBWV-2 based on the two ORFs’ nonrecombinant sequences. The expansion growth demographic model was supported as the best model for both ORF sequences based on a comparison of marginal likelihoods that were calculated using the harmonic-mean estimator in Tracer v 1.5.1. The relaxed-clock model provided a better fit than the strict-clock model, indicating the presence of rate variation among groups. Both ORF datasets of BBWV2 passed the date-randomization tests [50,51] and even met the more conservative criterion proposed by Duchêne et al. (2015) (Supplementary Materials Figure S2). These results suggest the presence of an adequate temporal signal in the two datasets. The mean evolutionary rates of the two ORF sequences were 7.828 × 10^−4^ subs/site/year (95% HPD 1.620 × 10^−3^–5.669 × 10^−5^) and 1.840 × 10^−3^ subs/site/year (95% HPD 3.267 × 10^−3^–4.517 × 10^−4^), respectively. The time to the most recent common ancestors (TMRCAs) was 471 years (101–1095) (Supplementary Materials Figure S1A) and 172 years (63–493) (Supplementary Materials Figure S1B) for the ORF1 and ORF2 coding sequences, with effective sample size (ESS) 957 and 561, respectively.

### 3.4. Nucleotide Bias Analysis

The nucleotide compositions of the two ORF sequences were calculated to assess the influence of compositional constraints on BBWV-2′s codon usage. Nucleotides U and A were most abundant with a mean composition of 28.42 ± 0.29% and 28.27 ± 0.20% (Supplementary Materials Table S3) for the ORF1 sequences, respectively, compared with G (26.21 ± 0.22%) and C (17.10 ± 0.25%). Similarly, nucleotides U (29.06 ± 0.34%) and A (27.94 ± 0.35%) were also most abundant in the ORF2 coding sequences, followed by G (24.28 ± 0.34%) and C (18.71 ± 0.29%) (Supplementary Materials Table S4). In terms of the third position’s nucleotide composition of synonymous codons, U_3S,_ A_3S_, G_3S_ and C_3S_ in both ORF sequences were consistent with the nucleotide composition despite the similar value of A_3S_ (33.13 ± 0.89%) and G_3S_ (33.64 ± 1.01%) in the ORF1 sequences (Supplementary Materials Tables S3 and S4). In addition, the composition of AU (56.69 ± 0.27% and 57.00 ± 0.42%) was also higher than that of GC (43.31 ± 0.27% and 43.00 ± 0.42%) in both ORF sequences of BBWV-2 (Supplementary Materials Tables S3 and S4). In all, these results suggest an AU-rich composition of BBWV-2 coding sequences.

An RSCU analysis was performed to estimate the codon usage pattern of the ORF1 and ORF2 coding sequences of BBWV-2 (Table 2). For the ORF1 coding sequences, 14 of 18 preferred codons were A/U-ended (both A- and U-ended: 7) (Table 2), and 16 of 18 preferred codons were A/U-ended (A-ended: 6; U-ended: 10) in the ORF2 coding sequences (Table 2). These results suggest that A- and U-ended codons were preferred in the BBWV-2 coding sequences. Within these preferred codons, three had a RSCU value > 1.6, with the highest being UUG for both the ORF1 (2.82) and ORF2 (2.66) coding sequences of BBWV-2, indicating extreme over-presentation. The remaining preferred codons of RSCU values were all more than 0.6 and smaller than 1.6. Moreover, no optional synonymous codons were under-represented (RSCU < 0.6) from the BBWV-2 coding sequences. In addition, the RSCU values of the BBWV-2 coding sequences in terms of hosts also indicated that A/U-ended codons were more frequent than G/C-ended codons (Table 2).

### 3.5. Codon Usage Bias of BBWV-2

ENC values were determined to show the magnitude of the two BBWV-2 ORF codon usage choices. Similar mean ENC values were observed for the two ORF coding sequences (51.48 ± 0.91 and 51.93 ± 0.95) (Supplementary Materials Tables S3 and S4). For ORF1, the highest mean ENC value of BBWV-2 was found in spinach while the lowest was in yam (Supplementary Materials Figure S3A). Meanwhile, the highest mean ENC value was found in pepper for ORF2 sequences (Supplementary Materials Figure S3B). The mean ENC values of the two ORF coding sequences were more than 35 (Supplementary Materials Tables S3 and S4) (Supplementary Materials Figure S3), indicating a conserved and stable genomic composition in the BBWV2 coding sequences.

#### 3.5.1. Trends in Codon Usage Variations

**Figure 3:**
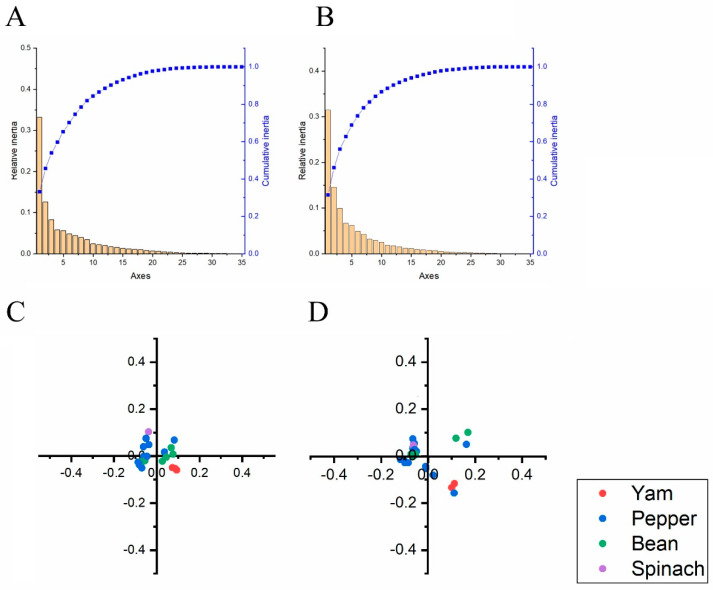
The relative and cumulative inertia of the 35 axes from a COA of the RSCU values based on the ORF1 (**A**) and ORF2 (**B**) sequences of broad bean wilt virus 2. PCA based on the RSCU values of the ORF1 (**C**) and ORF2 (**D**) sequences of broad bean wilt virus 2. The broad bean pepper, spinach and yam hosts are showed in green, blue, purple and red dots, respectively.

To investigate the synonymous codon usage variation in the two ORF sequences of BBWV-2, we performed a principal component analysis. The first four principal axes (axes 1–4) for the two ORF sequences of BBWV-2 accounted more than 50% (Figure 3A,B). The results also showed that Axis 1 was the major factor affecting codon usage of the two ORF sequences for BBWV-2. We also explored the distribution of the two ORF sequences in different hosts according to the RSCU values on the first two axes. Overlapping among the different hosts for the two BBWV-2 coding sequences was observed from the PCA analysis, indicating distinct codon usage trends (Figure 3C,D). However, only three yam and three spinach sequences were included in this analysis, so these results require further confirmation.

#### 3.5.2. ENC-Plot Analysis

**Figure 4:**
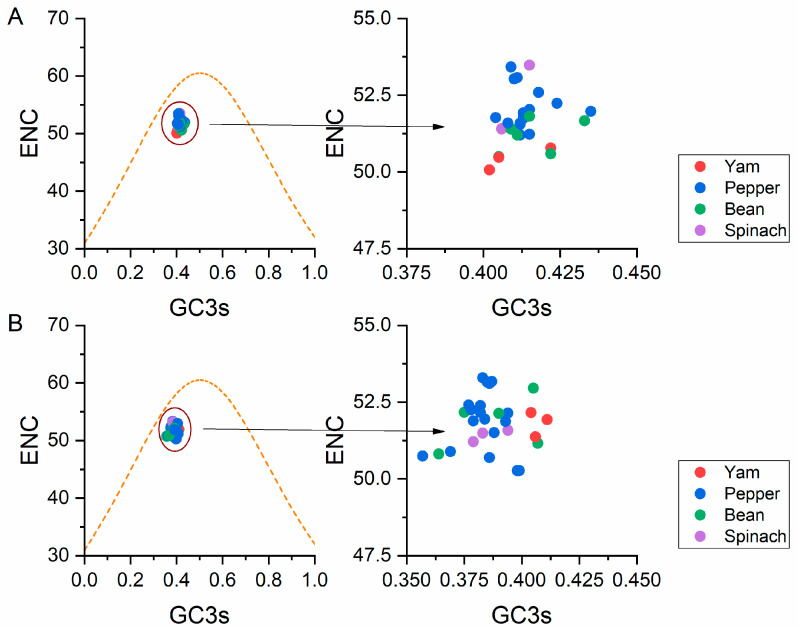
ENC-plot analysis of ORF1 (**A**) and ORF2 (**B**) sequences of broad bean wilt virus 2, with ENC against the GC3s of different hosts. The orange dotted line represents the standard curve when the codon usage bias is determined by the GC3s composition only. The broad bean pepper, spinach and yam hosts are showed in green, blue, purple and red dots, respectively.

A ENC-GC3s plot analysis was performed to assess the forces influencing the BBWV2 codon usage pattern. In general, points falling below the expected curve indicate that the codon usage is affected by natural selection rather than mutation. On the other hand, data points falling onto the expected curve which indicate mutational pressure. In the two plots of BBWV-2 sequences, all isolates regardless of host clustered together below the expected ENC curve (Figure 4), indicating that the influence of natural selection dominated that of mutation pressure.

#### 3.5.3. Neutrality Plot

**Figure 5:**
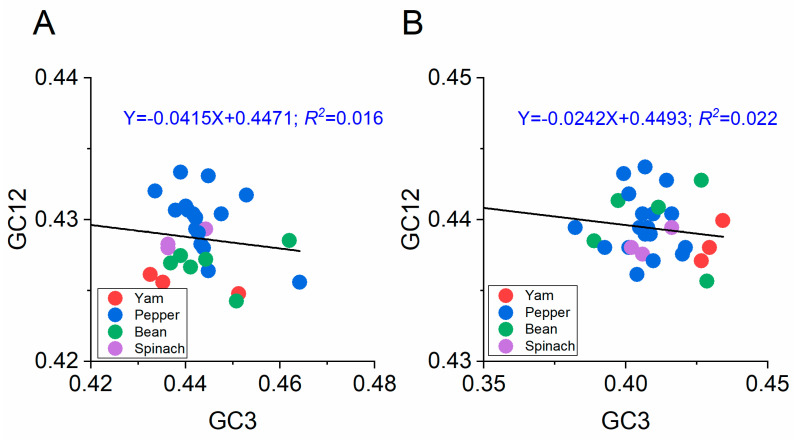
Neutrality plot analysis of GC3 against GC12 for the ORF1 (**A**) and ORF2 (**B**) sequences of broad bean wilt virus 2. The broad bean, pepper, spinach and yam hosts are represented in green, blue, purple and red dots, respectively.

The influence of mutation and natural selection on BBWV-2 codon usage was assessed using a neutrality analysis (Figure 5). Generally, nucleotide changes at the third position of the codon do not influence the changes in amino acids, so they are considered only a mutational force. Meanwhile, a nucleotide change causing a change in amino acid is considered a selection force. Among the two ORF sequences, a negative correlation was observed between the GC12 and GC3 values for BBWV-2 (Figure 5). The slopes of the linear regression were −0.0415 and −0.0242 for the ORF1 and ORF2 coding sequences (Figure 5), respectively. These results indicate that mutation pressure accounted for 4.15% and 2.42% of the selection force for the ORF1 and ORF2 coding sequences, whereas natural selection accounted for 95.85% and 97.58%, respectively. Thus, the neutrality analysis indicated that natural selection dominated the forces shaping the codon usage pattern of BBWV-2.

#### 3.5.4. Parity Analysis

**Figure 6:**
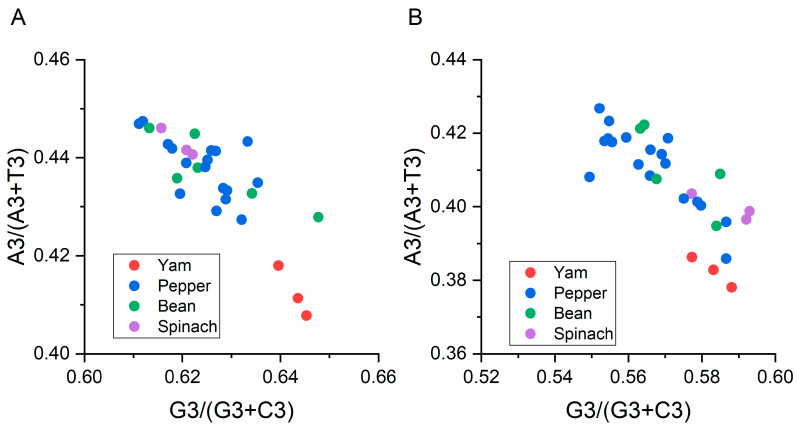
Parity plot showing the presence of AT bias [A3%/(A3% + T3%)] and GC bias [G3%/(G3% + C3%)] for the ORF1 (**A**) and ORF2 (**B**) sequences of broad bean wilt virus 2. The center of the plot, where the value of both coordinates is 0.5, indicates the place where there is no bias in mutation or selection rates. The broad bean pepper, spinach and yam hosts are represented in green, blue, purple and red dots, respectively.

To assess whether highly biased genes exhibited biased codon selection in the two BBWV2 coding regions, we performed a PR2 bias plot analysis. Generally, the centre of the plot (A = T and G = C) is the place where both coordinates are 0.5, and it is also the place where no bias is present in the selection (substitution rates) or mutation force [12]. Here, the nucleotides A and C are less commonly used than U and G in the two BBWV-2 coding sequences (Figure 6). These give a novel perspective on the genetic divergence of BBWV-2 and explore factors shaping its codon usage patterns.

Furthermore, to calculate the influence of natural selection pressure on BBWV-2 codon usage bias, a linear regression analysis between the ARO and GRAVY values and GC3S, GC, ENC, and the first two principal axes values were also performed here. The correlation analysis based on ORF1 sequences showed that GRAVY is significantly negatively correlated with Axis 1. AROMO showed a significant positive correlation with ENC and a significant negative correlation with Axis 1 (Table 3). For the ORF2 sequences, our correlation analysis indicated that GRAVY is significantly negatively correlated with Axis 1 and Axis 2, and AROMO showed a significant negative correlation with ENC (Table 3). These results indicate that the general average aromaticity and hydropathicity are correlated to the codon usage variation in BBWV-2, indicating the influence of natural selection pressure on the BBWV-2′s codon usage pattern.

### 3.6. Codon Usage Adaptation in BBWV-2

**Figure 7:**
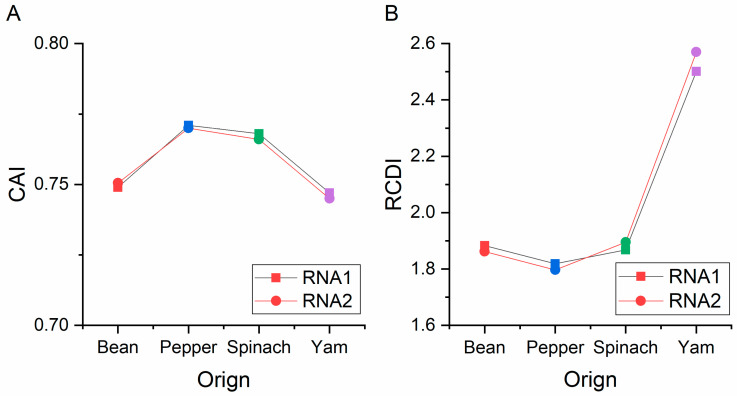
The CAI analysis and RCDI analysis of the ORF1 (**A**) and ORF2 (**B**) sequences of broad bean wilt virus 2 in relation to the natural hosts. The *x*-axis represents the sequences identified in different hosts.

**Figure 8:**
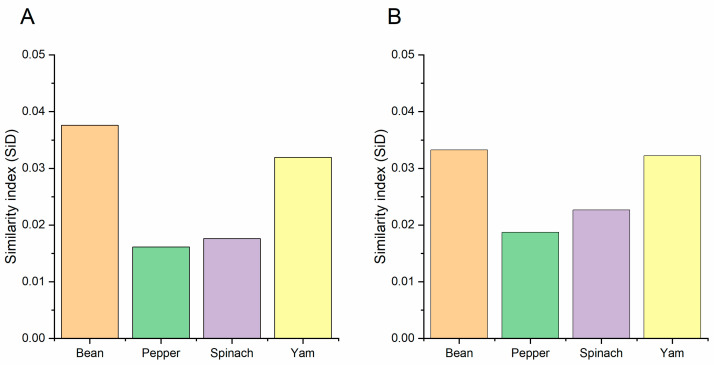
The SiD analysis of the ORF1 (**A**) and ORF2 (**B**) sequences of broad bean wilt virus 2 in relation to the natural hosts. The broad bean pepper, spinach and yam hosts are showed in light orange, green, purple and yellow column, respectively. The *x*-axis represents the sequences identified in different hosts.

CAI values were assessed to determine the adaptation and codon usage optimization of BBWV-2 to its hosts. Generally, sequences with higher CAI values are considered to be more adapted to hosts than sequences with low values. Here, the mean CAI values of the ORF1 sequences were 0.749, 0.771, 0.768 and 0.747 for broad bean, pepper, spinach and yam, respectively (Figure 7). The mean CAI values of the ORF2 coding sequences were 0.751, 0.770, 0.766 and 0.745 for the broad bean, pepper, spinach and yam, respectively (Figure 7). These results indicate that the BBWV-2 genes have codon usage preferences that are closer to pepper than to other hosts. Then, to show the cumulative effects of codon biases on a single gene’s expression, we also performed an RCDI analysis. The means of the RCDI values for both ORF sequences were highest for yam, followed by broad bean, spinach and pepper (Figure 7). These results also indicate that the BBWV-2 genes have codon usage preferences that are closer to pepper than to other hosts. Moreover, we performed an SiD analysis to understand how the codon usage patterns of broad bean, pepper, spinach and yam affect the BBWV-2 codon usage pattern (Figure 8). Among the two ORF coding sequences, the highest SiD values were all observed in broad bean (Figure 8) while the lowest values were found in pepper, although these SiD values for broad bean, pepper, spinach and yam were very low. These results indicate that during BBWV-2 evolution broad bean and pepper probably had a greater impact on the virus than spinach and yam.

## 4. Discussion

The evolutionary analysis and genetic variation of BBWV-2 from broad bean or pepper have been described based on analyses of partial or complete genome sequences [2,25]. In this study, our results provide significant insight into the evolutionary patterns of BBWV-2. For segmented viruses, rapid genomic changes were driven by both recombination and reassortment [58,59]. Previously, recombination was proven to be the factors shaping the evolution of BBWV-2 [2]. Here, our current findings suggest that genetic exchange by reassortment also had a significant influence on the genomic composition of BBWV-2.

A phylogenetic analysis of BBWV-2 showed six divergent evolutionary lineages based on partial genomic sequences [2]. In the present study, our phylogenetic analysis based on ML and MCC found three and four lineages based on the ORF1 and ORF2 protein coding sequences, respectively. The evolutionary rates of the two BBWV-2 ORF coding sequences were 8.895 × 10^−4^ and 4.560 × 10^−4^ subs/site/year, respectively, similar to tobacco mosaic virus (TMV) [60] but slightly slower than the previously reported plant RNA viruses such as PVM [61], turnip mosaic virus [62], odontoglossum ringspot virus (ORSV) [63], and potato virus Y [64].

The codon usage pattern has a significant influence on virus evolution, such as adaption, evolution, evasion from the host’s immune system, and survival [55,65,66,67,68]. Presently, only several reports have described the influence of codon usage in the evolution of plant viruses. Here, we firstly assessed the codon usage pattern and composition of BBWV2 based on the complete genome. Normally, AU-rich genomes tend to contain codons ending with A and U, while genomes with a GC-rich composition tend to contain codons ending with G and C. In this study, our nucleotide composition results show that codons ended with A and U are more frequent in BBWV-2 coding sequences. Generally, the preferred codons have been mostly determined by compositional constraints (A and U in this case in the two BBWV2 ORF coding regions), which also supports the presence of mutation pressure. And, the RSCU analysis also indicated that A/U-ended codons were more frequent than G/C-ended codons.

Normally, RNA viruses have low codon usage bias to perform efficient replication in the host by lowering the competition with the host genes [55,65,66,67,68]. Previous reports showed low codon usage bias from several plant viruses such as CTV, PRSV, PVM, RSV and SCMV [19,20,21,22,23,24]. In our study, a lower codon usage pattern of the BBWV2 genome (the ENC values higher than 35) was also found, indicating a low degree of preference. The ENC-plot, PR2, neutrality plot and regression analyses between the ARO and GRAVY values and ENC, GC, GC3S, and the first two principle axes values significantly showed that BBWV-2 is influenced by natural selection and mutation pressure to variable degrees. Consistent with PVM [23], both the ENC-plot and neutrality plot analyses showed that natural selection is the key factor shaping the codon usage pattern of BBWV-2.

The evolution and dynamics of infectious diseases are influenced by host–parasite interactions [69,70,71]. For viruses, several reports showed that codon usage patterns have a significant effect on the viruses’ host-specific adaption [23,55,67,68]. In this study, our CAI analysis showed that both BBWV-2 genes have codon usage preferences that are closer to pepper than to other hosts. In addation, the RCDI analysis showed that the lowest codon usage deoptimization also occurred for the BBWV-2 isolates for pepper followed by spinach. Generally, low RCDI values indicate strong adaptation to a host [72]. Thus, both CAI and RCDI analyses were consistent supported that BBWV-2 were most strongly adapted to pepper than broad bean, spinach and yam. However, the SiD value for the BBWV-2 isolates from broad bean was higher than those observed for yam, spinach and pepper, indicating that the selection pressure of broad bean on BBWV-2 isolates was greater than that of yam, spinach and pepper, possibly due to BBWV-2 originated from broad bean.

In conclusion, the codon usage patterns and host adaptability of BBWV-2 were studied for the first time to investigate its evolutionary changes. Reassortment also had a significant influence on the genomic evolution of BBWV-2. The ENC-plot and neutrality plot analyses showed that natural selection is the key factor shaping the codon usage pattern of BBWV-2. A strong correlation between BBWV-2 and both broad bean and pepper was observed from the CAI, RCDI and SiD analyses. Our study furthers the understanding of the evolutionary changes of BBWV-2, which may provide a better understanding of the origin of BBWV-2.

## Figures and Tables

**Table 1:** Recombination sites detected in the protein encoding regions of broad bean wilt virus 2.

Segment	Isolate	Sequence Used to Infer Major Parent	Sequence Used to Infer Minor Parent	Recombination Site ^a^	Recombination Detecting Program (*p*-Value ^b^)						
					RDP	GENECONV	BOOTSCAN	MAXCHI	CHIMAERA	SISCAN	3SEQ
RNA1	KF498696	UN ^c^	FN985164	1311–2836	2.399 × 10^−7^	7.723 × 10^−1^	4.485 × 10^−8^	5.120 × 10^−5^	3.670 × 10^−2^	2.467 × 10^−11^	1.869 × 10^−6^
	KM076648	KC625492	AB023484	1644–5592	2.711 × 10^−141^	9.401 × 10^−134^	5.393 × 10^−133^	7.798 × 10^−44^	1.023 × 10^−30^	1.281 × 10^−55^	8.626 × 10^−13^
RNA2	JQ855708	KJ825857	KC625506	236–1144	2.057 × 10^−26^	1.165 × 10^−19^	9.826 × 10^−25^	1.522 × 10^−16^	5.628 × 10^−14^	1.524 × 10^−33^	1.096 × 10^−12^
	KM076649	JX183234	KJ825857	148–3166	3.811 × 10^−23^	1.442 × 10^−15^	6.082 × 10^−20^	1.152 × 10^−7^	1.394 × 10^−6^	1.938 × 10^−9^	2.193 × 10^−12^
	HQ283389	KJ825857	KC625506	236–1132	1.132 × 10^−30^	3.734 × 10^−22^	8.940 × 10^−29^	7.192 × 10^−22^	5.962 × 10^−16^	2.644 × 10^−26^	1.096 × 10^−12^
	KF498697	KC625506	LC497425	2561–3166	6.698 × 10^−8^	-	3.281 × 10^−8^	5.828 × 10^−6^	1.533 × 10^−2^	5.191 × 10^−12^	8.114 × 10^−4^
	GQ202215	KF498697	KX686590	1484–2576	5.190 × 10^−13^	1.640 × 10^−8^	3.624 × 10^−15^	6.479 × 10^−13^	4.696 × 10^−6^	3.525 × 10^−22^	1.096 × 10^−12^
	HQ283390	AB018698	LC497425	1278–3166	6.174 × 10^−22^	9.652 × 10^−18^	1.257 × 10^−12^	8.796 × 10^−23^	5.680 × 10^−12^	1.397 × 10^−16^	4.858 × 10^−40^

^a^ Recombination sites correspond to the two coding sequences of BBWV-2 Zhejiang isolate (GenBank accession number, RNA1, NC_003003; RNA2, NC_003004). ^b^ The analyses were done using default settings and a Bonferroni-corrected *p*-values cut-off of 0.01 in RDP4 software. ^c^ UN, Unknown.

**Table 2:** The RSCU value of 59 codons encoding 18 amino acids according to hosts of BBWV-2 ORF1 and ORF2.

Codon	aa	ORF1					ORF2				
		Broad Bean (*n* = 6)	Pepper (*n* = 18)	Spinach (*n* = 3)	Yam (*n* = 4)	All (*n* = 42)	Broad Bean (*n* = 5)	Pepper (*n* = 18)	Spinach (*n* = 3)	Yam (*n* = 4)	All (*n* = 41)
TTT	F	1.38 *	**1.37**	**1.38**	**1.35**	**1.35**	**1.25**	**1.35**	**1.28**	**1.25**	**1.32**
TTC	F	0.62	0.63	0.62	0.65	0.65	0.75	0.65	0.73	0.76	0.68
TTA	L	0.76	0.80	0.76	0.71	0.79	0.73	0.72	0.58	0.66	0.72
TTG	L	**2.97**	**2.72**	**2.86**	**3.02**	**2.82**	**2.66**	**2.50**	**2.77**	**2.81**	**2.66**
CTT	L	0.95	0.94	0.85	1.13	0.96	1.20	1.30	1.35	1.20	1.21
CTC	L	0.42	0.45	0.48	0.44	0.44	0.31	0.27	0.20	0.25	0.31
CTA	L	0.29	0.32	0.31	0.22	0.31	0.31	0.33	0.29	0.32	0.33
CTG	L	0.61	0.77	0.74	0.47	0.68	0.80	0.79	0.82	0.75	0.77
ATT	I	**1.50**	**1.53**	**1.53**	**1.60**	**1.54**	**1.40**	**1.35**	**1.27**	**1.43**	**1.36**
ATC	I	0.64	0.57	0.61	0.54	0.59	0.62	0.69	0.72	0.72	0.68
ATA	I	0.86	0.89	0.86	0.86	0.87	0.98	0.96	1.01	0.84	0.96
GTT	V	1.33	1.38	1.37	1.22	1.37	1.32	1.19	1.29	1.28	1.29
GTC	V	0.58	0.57	0.58	0.70	0.57	0.56	0.67	0.54	0.61	0.63
GTA	V	0.38	0.34	0.45	0.40	0.36	0.54	0.55	0.58	0.50	0.51
GTG	V	**1.71**	**1.71**	**1.61**	**1.69**	**1.7**	**1.58**	**1.59**	**1.59**	**1.62**	**1.57**
TCT	S	1.07	1.15	1.15	1.15	1.12	1.26	1.27	**1.51**	1.2	1.28
TCC	S	0.54	0.59	0.58	0.41	0.54	0.58	0.62	0.47	0.65	0.6
TCA	S	**1.70**	**1.46**	**1.60**	**1.66**	**1.57**	1.23	1.23	1.19	1.03	1.19
TCG	S	0.55	0.64	0.52	0.63	0.61	0.35	0.30	0.32	0.46	0.35
AGT	S	1.18	1.05	1.00	1.36	1.14	**1.50**	**1.53**	1.46	**1.58**	**1.53**
AGC	S	0.96	1.11	1.16	0.79	1.02	1.09	1.05	1.06	1.08	1.05
CCT	P	1.38	1.14	1.29	**1.57**	1.28	**1.69**	**1.71**	**1.76**	**1.30**	**1.66**
CCC	P	0.77	0.85	0.74	0.66	0.78	0.70	0.67	0.51	1.03	0.72
CCA	P	**1.43**	**1.62**	**1.44**	1.51	**1.54**	1.17	1.23	1.22	0.98	1.18
CCG	P	0.42	0.39	0.53	0.26	0.39	0.43	0.38	0.51	0.69	0.44
ACT	T	1.27	1.25	1.23	1.33	1.26	1.41	**1.43**	**1.42**	**1.64**	**1.44**
ACC	T	0.53	0.48	0.47	0.51	0.49	0.52	0.49	0.49	0.30	0.47
ACA	T	**1.57**	**1.63**	**1.71**	**1.49**	**1.59**	**1.50**	1.42	1.38	1.50	1.43
ACG	T	0.64	0.64	0.59	0.66	0.65	0.58	0.66	0.72	0.56	0.65
GCT	A	1.32	1.31	1.28	**1.59**	1.37	1.26	1.32	**1.36**	**1.37**	1.32
GCC	A	0.70	0.72	0.77	0.57	0.69	0.62	0.62	0.59	0.79	0.64
GCA	A	**1.47**	**1.40**	**1.39**	1.34	**1.4**	**1.5**	**1.45**	1.34	1.26	**1.45**
GCG	A	0.51	0.57	0.56	0.50	0.54	0.62	0.61	0.71	0.58	0.58
TAT	Y	**1.12**	**1.12**	**1.18**	**1.14**	**1.14**	**1.09**	**1.06**	**1.11**	0.99	**1.05**
TAC	Y	0.88	0.88	0.82	0.86	0.86	0.91	0.94	0.89	**1.01**	0.95
CAT	H	**1.42**	**1.40**	**1.33**	**1.51**	**1.46**	**1.46**	**1.45**	**1.52**	**1.35**	**1.39**
CAC	H	0.58	0.60	0.67	0.49	0.54	0.54	0.55	0.49	0.65	0.61
CAA	Q	**1.10**	**1.10**	**1.04**	**1.11**	**1.10**	**1.26**	**1.20**	**1.17**	**1.37**	**1.23**
CAG	Q	0.90	0.90	0.96	0.89	0.92	0.74	0.80	0.83	0.63	0.77
AAT	N	**1.36**	**1.37**	**1.40**	**1.30**	**1.35**	**1.37**	**1.38**	**1.45**	**1.48**	**1.39**
AAC	N	0.65	0.63	0.60	0.70	0.65	0.63	0.62	0.55	0.52	0.61
AAA	K	0.96	0.97	0.98	0.86	0.95	**1.09**	**1.09**	**1.03**	**1.01**	**1.06**
AAG	K	**1.04**	**1.03**	**1.02**	**1.14**	**1.05**	0.91	0.91	0.97	0.99	0.94
GAT	D	**1.48**	**1.54**	**1.53**	**1.48**	**1.51**	**1.53**	**1.53**	**1.54**	**1.54**	**1.54**
GAC	D	0.52	0.46	0.47	0.52	0.49	0.47	0.47	0.46	0.46	0.46
GAA	E	0.88	0.94	**1.02**	0.93	0.93	1.00	**1.05**	**1.03**	0.73	**1.01**
GAG	E	**1.12**	**1.06**	0.99	**1.07**	**1.07**	**1.01**	0.95	0.97	**1.27**	0.99
TGT	C	**1.06**	1.00	**1.04**	**1.12**	**1.04**	**1.38**	**1.31**	**1.08**	**1.69**	**1.29**
TGC	C	0.94	**1.01**	0.96	0.88	0.96	0.62	0.69	0.92	0.31	0.71
CGT	R	0.54	0.52	0.56	0.72	0.57	1.30	1.19	1.09	1.13	1.15
CGC	R	0.67	0.79	0.82	0.52	0.69	1.14	1.29	1.30	0.99	1.2
CGA	R	0.59	0.58	0.70	0.39	0.59	0.29	0.31	0.25	0.35	0.36
CGG	R	0.47	0.55	0.42	0.55	0.49	0.21	0.14	0.21	0.12	0.16
AGA	R	**2.03**	**2.04**	**2.03**	1.88	**1.97**	**2.16**	**2.45**	**2.64**	**1.91**	**2.3**
AGG	R	1.69	1.52	1.47	**1.95**	1.69	0.90	0.62	0.50	1.51	0.82
GGT	G	0.89	1.10	1.11	1.13	1.04	1.08	1.12	1.16	**1.52**	1.18
GGC	G	0.84	0.73	0.79	0.65	0.75	0.78	0.73	0.73	0.64	0.74
GGA	G	**1.56**	**1.46**	**1.45**	**1.51**	**1.49**	**1.29**	**1.32**	**1.29**	1.20	**1.32**
GGG	G	0.71	0.72	0.65	0.72	0.72	0.84	0.83	0.812	0.65	0.77

* The optimal RSCU values are shown in bold.

**Table 3:** Correlation analysis among GRAVY, ARO, ENC, GC3_S_, GC, and the first two principle axes.

Gene		ENC		GC3s		GC		Axis1		Axis2	
		r	*p*	r	*p*	r	*p*	r	*p*	r	*p*
ORF1	Gravy	0.13091 *^ns^*	0.40274	0.01371 *^ns^*	0.93045	0.05954 *^ns^*	0.7045	−0.32221 *	0.0351	−0.25199 *^ns^*	0.10307
	Aromo	0.47618 **	0.00125	−0.21277 *^ns^*	0.17073	0.26115 *^ns^*	0.09074	−0.85596 **	2.58 × 10^−13^	0.03121 *^ns^*	0.8425
ORF2	Gravy	0.02381 *^ns^*	0.88105	0.25202 *^ns^*	0.10738	0.20002 *^ns^*	0.20408	−0.43133 **	0.00435	−0.37632 *	0.01404
	Aromo	−0.38519 *	0.01177	−0.10812 *^ns^*	0.49553	−0.22767 *^ns^*	0.14705	−0.27126 *^ns^*	0.08227	−0.27379 *^ns^*	0.07934

*^ns^* non-significant (*p* > 0.05); * represents 0.01 < *p* < 0.05; ** represents *p* < 0.01.

## Data Availability

All data presented in this study are available on request from the corresponding authors.

## Supplementary Materials

The following are available online at https://www.mdpi.com/1999-4915/13/2/198/s1, Figure S1. Bayesian maximum-clade-credibility tree inferred from trees calculated from the ORF1 (A) and ORF2 (B) sequences of broad bean wilt virus 2. Horizontal blue bars represent the 95% credibility intervals of the estimates of node ages. The tree topology was chosen to maximize the product of node posterior probabilities. Only posterior probability values above 0.95 are shown. Year before present; 2017. Figure S2. Estimates of nucleotide substitution rates. Mean estimates and the 95% highest posterior density interval (HPD) are shown. They were estimated from the polyprotein sequences of nonrecombinant sugarcane mosaic virus. The first value is based on the original data, whereas the remaining ten values are from date-randomized replicates in each set of estimates. The 95% HPD of the estimates from the date-randomized replicates did not overlap with the mean posterior estimate from the original data set. Moreover, the lower tails of the credibility intervals were long and tended towards zero. These features suggest sufficient temporal structure in the original data sets for rate estimation. Figure S3 ENC values for the ORF1 (A) and ORF2 (B) sequences of broad bean wilt virus 2. Table S1: The BBWV2 isolates using in this study, Table S2. Reassortment analysis by RDP software using 38 BBWV2 artificially concatenated sequences. Table S3. Codon data of broad bean wilt virus 2 ORF1. Table S4. Codon data of broad bean wilt virus 2 ORF2. References [26,27,28,29,73,74,75,76] are cited in the supplementary materials.
