# Supplementary material for: Phylodynamics and Codon Usage Pattern Analysis of Broad Bean Wilt Virus 2
Source: Viruses. 2021 Jan 28;13(2):198. doi: 10.3390/v13020198 (PMC7912035; doi:10.3390/v13020198)
Supplement: Supplementary file 1 [file viruses-13-00198-s001.zip › viruses-1048063-supppconv.pdf]

viruses

MDPI

Supplementary Materials: Phylodynamics and Codon Usage Pattern Analysis of Broad Bean Wilt Virus 2

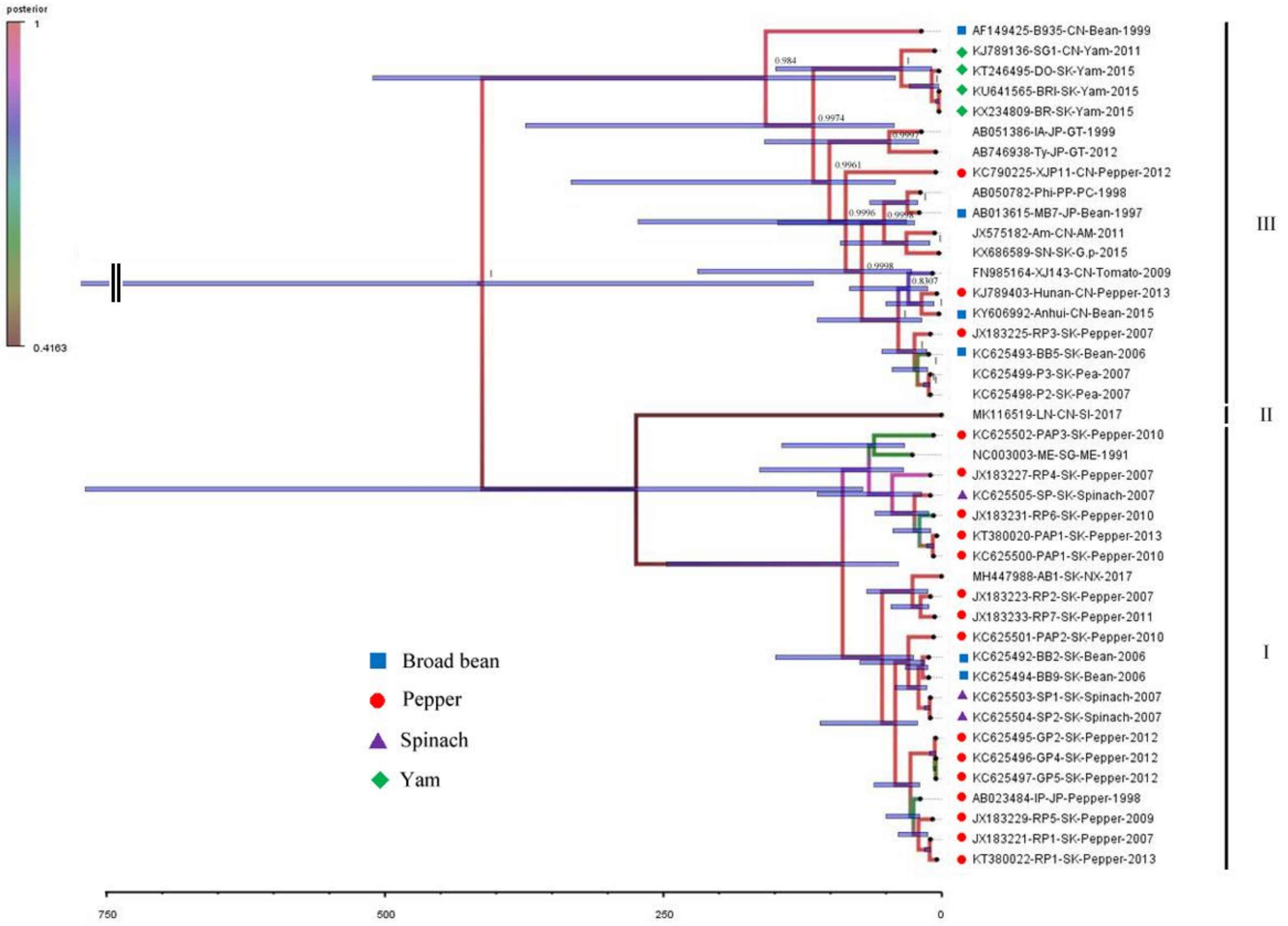

A

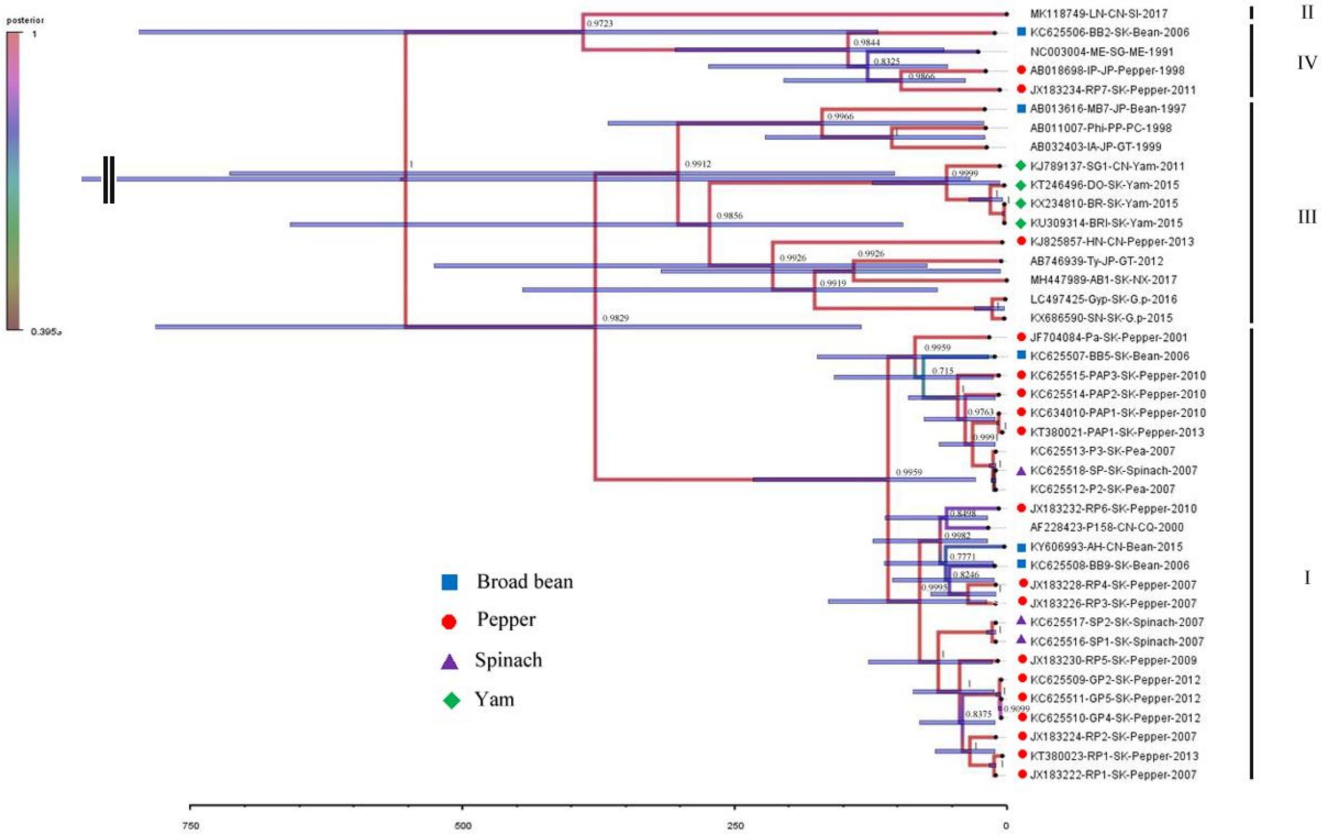

B

Figure S1. Bayesian maximum-clade-credibility tree inferred from trees calculated from the ORF1 (**A**) and ORF2 (**B**) sequences of broad bean wilt virus 2. Horizontal blue bars represent the 95% credibility intervals of the estimates of node ages. The tree topology was chosen to maximize the product of node posterior probabilities. Only posterior probability values above 0.95 are shown. Year before present; 2017.

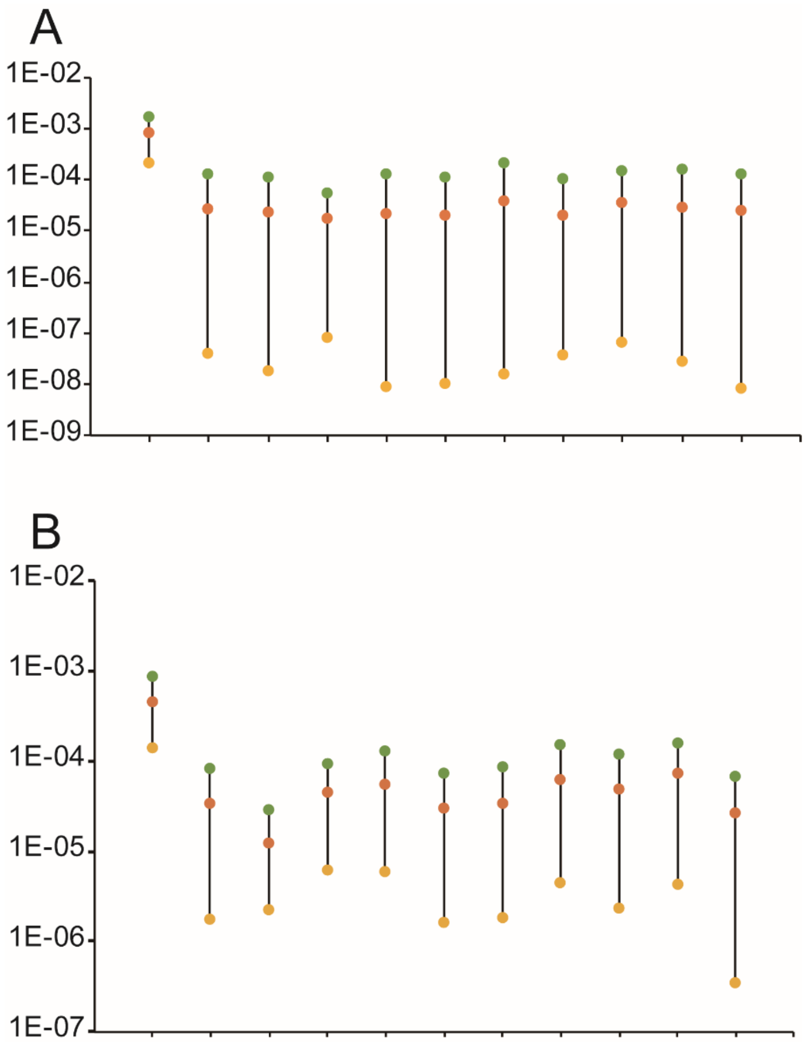

Figure S2. Estimates of nucleotide substitution rates. Mean estimates and the 95% highest posterior density interval (HPD) are shown. They were estimated from the polyprotein sequences of nonrecombinant sugarcane mosaic virus. The first value is based on the original data, whereas the remaining ten values are from date-randomized replicates in each set of estimates. The 95% HPD of the estimates from the date-randomized replicates did not overlap with the mean posterior estimate from the original data set. Moreover, the lower tails of the credibility intervals were long and tended towards zero. These features suggest sufficient temporal structure in the original data sets for rate estimation..

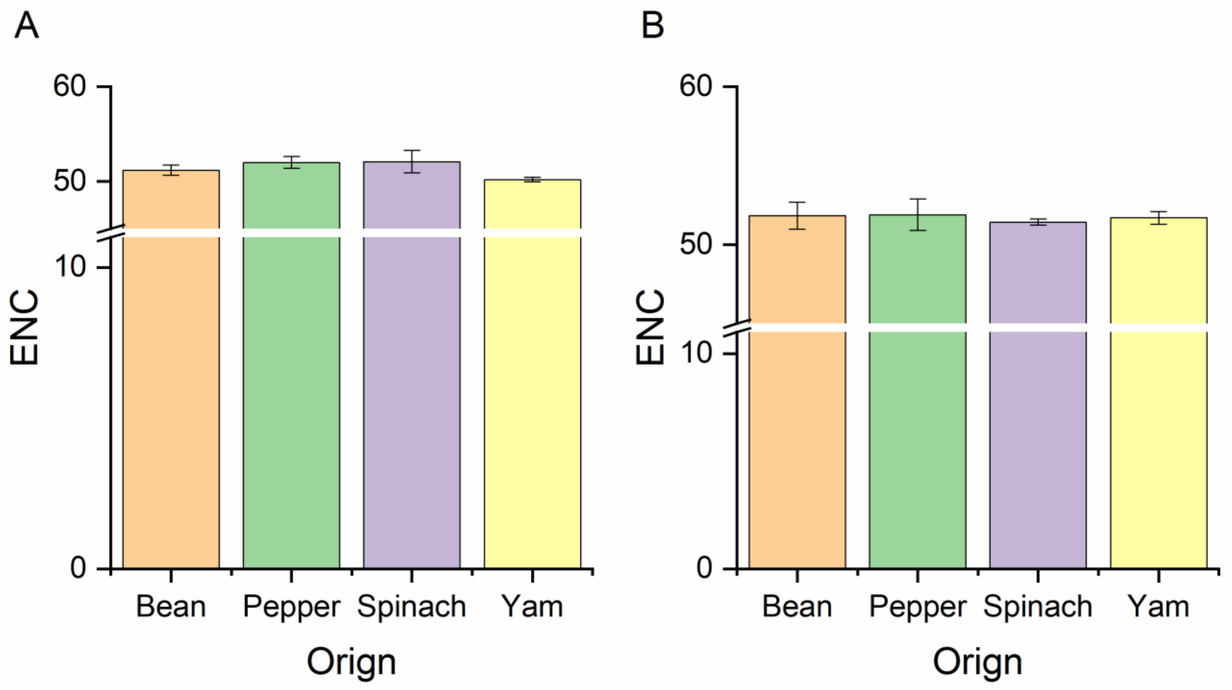

Figure S3 ENC values for the ORF1 (**A**) and ORF2 (**B**) sequences of broad bean wilt virus 2.

Table S1. The BBWV2 isolates using in this study.

Accession no	Geographic origin	Host	Time	Isolate	Segment	Length	References
KJ789137	China: Shandong	*Dioscorea opposita*	2011	SG1	RNA2	3599	
KJ789136	China: Shandong	*Dioscorea opposita*	2011	SG1	RNA1	5953	
KC790225	China: Xinjiang	Pepper	2012	XJP1-1	RNA1	5958	
JQ855708	China: Xinjiang	Tomato	2009	XJ14-3a	RNA2	3599	
JF704084	South Korea	Pepper	2001	Pa	RNA2	3596	
MH447989	South Korea: Naju	*Achyranthes bidentata*	2017	AB1	RNA2	3597	
MH447988	South Korea: Naju	*Achyranthes bidentata*	2017	AB1	RNA1	5950	
NC003004	Singapore	*Megakepasma erythrochlamys*	1991	ME	RNA2	3607	[26]
NC003003	Singapore	*Megakepasma erythrochlamys*	1991	ME	RNA1	5951	[26]
KY606993	China: Anhui	*Vicia faba*	2015	Anhui	RNA2	3586	
KY606992	China: Anhui	*Vicia faba*	2015	Anhui	RNA1	5943	
KX234809	South Korea: Andong	*Dioscorea opposita*	2015	BR	RNA1	5955	
KX686590	South Korea	*Gynura procumbens*	2015	SN	RNA2	3601	
KX686589	South Korea	*Gynura procumbens*	2015	SN	RNA1	5958	
KU309314.	South Korea: Andong	*Dioscorea opposita*	2015	BRI	RNA2	3599	
KU641565.	South Korea: Andong	*Dioscorea opposita*	2015	BRI	RNA1	5955	
KT246496	South Korea	*Dioscorea opposita*	2015	DO	RNA2	3598	
KT246495	South Korea	*Dioscorea opposita*	2015	DO	RNA1	5954	
AF149425	China:Zhejiang	*Vicia faba*	<1999	B935	RNA1	5956	[29]
KT380023	South Korea	*Capsicum annuum*	2013	RP1	RNA2	3595	[27]
KT380022	South Korea	*Capsicum annuum*	2013	RP1	RNA1	5952	[27]
KT380021	South Korea	*Capsicum annuum*	2013	PAP1	RNA2	3603	[27]
KT380020	South Korea	*Capsicum annuum*	2013	PAP1	RNA1	5952	[27]
JX183234	South Korea	*Capsicum annuum*	2011	RP7	RNA2	3571	
JX183233	South Korea	*Capsicum annuum*	2011	RP7	RNA1	5950	
JX183232	South Korea	*Capsicum annuum*	2010	RP6	RNA2	3595	
JX183231	South Korea	*Capsicum annuum*	2010	RP6	RNA1	5955	
JX183230	South Korea	*Capsicum annuum*	2009	RP5	RNA2	3594	
JX183229	South Korea	*Capsicum annuum*	2009	RP5	RNA1	5952	
JX183228	South Korea	*Capsicum annuum*	2007	RP4	RNA2	3596	
JX183226	South Korea	*Capsicum annuum*	2007	RP3	RNA2	3596	
JX183225	South Korea	*Capsicum annuum*	2007	RP3	RNA1	5955	

JX183224	South Korea	*Capsicum annuum*	2007	RP2	RNA2	3596	
JX183223	South Korea	*Capsicum annuum*	2007	RP2	RNA1	5950	
JX183222	South Korea	*Capsicum annuum*	2007	RP1	RNA2	3595	
JX183221	South Korea	*Capsicum annuum*	2007	RP1	RNA1	5952	
KM076649	South Korea: Andong	*Leonurus sibiricus*	2012	LS2	RNA2	3575	[73]
KM076648	South Korea: Andong	*Leonurus sibiricus*	2012	LS2	RNA1	5951	[73]
KJ825857	China: Hunan	*Capsicum annuum*	2013	Hunan	RNA2	3609	
KJ789403	China: Hunan	*Capsicum annuum*	2013	Hunan	RNA1	5956	
KC625518	South Korea	*Spinacia oleracea*	2007	SP	RNA2	3603	
KC625517	South Korea	*Spinacia oleracea*	2007	SP2	RNA2	3596	
KC625516	South Korea	*Spinacia oleracea*	2007	SP1	RNA2	3596	
KC625515	South Korea	*Capsicum annuum*	2010	PAP3	RNA2	3602	
KC625514	South Korea	*Capsicum annuum*	2010	PAP2	RNA2	3604	
KC625513	South Korea	*Pisum sativum*	2007	P3	RNA2	3603	
KC625512	South Korea	*Pisum sativum*	2007	P2	RNA2	3603	
KC625511	South Korea	*Capsicum annuum*	2012	GP5	RNA2	3596	
KC625510	South Korea	*Capsicum annuum*	2012	GP4	RNA2	3596	
KC625509	South Korea	*Capsicum annuum*	2012	GP2	RNA2	3596	
KC625508	South Korea	*Vicia faba*	2006	BB9	RNA2	3595	
KC625507	South Korea	*Vicia faba*	2006	BB5	RNA2	3594	
KC625506	South Korea	*Vicia faba*	2006	BB2	RNA2	3568	
KC625505	South Korea	*Spinacia oleracea*	2007	SP	RNA1	5950	
KC625504	South Korea	*Spinacia oleracea*	2007	SP2	RNA1	5951	
KC625503	South Korea	*Spinacia oleracea*	2007	SP1	RNA1	5951	
KC625502	South Korea	*Capsicum annuum*	2010	PAP3	RNA1	5951	
KC625501	South Korea	*Capsicum annuum*	2010	PAP2	RNA1	5952	
KC625500	South Korea	*Capsicum annuum*	2010	PAP1	RNA1	5952	
KC625499	South Korea	*Pisum sativum*	2007	P3	RNA1	5955	
KC625498	South Korea	*Pisum sativum*	2007	P2	RNA1	5955	
KC625497	South Korea	*Capsicum annuum*	2012	GP5	RNA1	5952	
KC625496	South Korea	*Capsicum annuum*	2012	GP4	RNA1	5952	
KC625495	South Korea	*Capsicum annuum*	2012	GP2	RNA1	5952	
KC625494	South Korea	*Vicia faba*	2006	BB9	RNA1	5953	
KC625493	South Korea	*Vicia faba*	2006	BB5	RNA1	5956	
KC625492	South Korea	*Vicia faba*	2006	BB2	RNA1	5952	
KC634010	South Korea	*Capsicum annuum*	2010	PAP1	RNA2	3603	

HQ283390	China: Xinjiang	*Capsicum annuum*	2012	XJP1-1	RNA2	3616	
HQ283389	China: Xinjiang	Tomato	2009	XJ14-3	RNA2	3635	
AB013616	Japan	*Vicia fava*	1997	MB7	RNA2	3589	[28]
AB013615	Japan	*Vicia fava*	1997	MB7	RNA1	5957	[28]
LC497425	South Korea: Gangwon-do	*Gynura procumbens*	2016	Gyp	RNA2	3601	
KX234810	South Korea: Andong	*Dioscorea opposita*	2015	BR	RNA2	3599	
JX183227	South Korea	*Capsicum annuum*	2007	RP4	RNA1	5952	
KF498697	China:Shanxi	*Capsicum annuum*	2012	Ca	RNA2	3559	
KF498696	China:Shanxi	*Capsicum annuum*	2012	Ca	RNA1	5929	
AB746939	Japan	*Gentiana triflora*	<2012	Ty	RNA2	3600	[74]
AB746938	Japan	*Gentiana triflora*	<2012	Ty	RNA1	5955	[74]
KC110085	China:Shanxi	*Atractylodes macrocephala*	2011	Am	RNA2	3548	
JX575182	China:Shanxi	*Atractylodes macrocephala*	2011	Am	RNA1	5949	
GQ202215	China	*Rehmannia glutinosa*	2008	DH	RNA2	3525	
AB051386	Japan	*Gentiana triflora*	<1999	IA	RNA1	5955	[75]
AB032403	Japan	*Gentiana triflora*	<1999	IA	RNA2	3593	[75]
AB023484	Japan	*Capsicum annuum*	<1998	IP	RNA1	5953	
MK116519	China:Liaoning	*Sesamum indicum*	2017	LN	RNA1	5955	
MK118749	China:Liaoning	*Sesamum indicum*	2017	LN	RNA2	3573	
AF104335	South Korea	Unknown	<1998	KK	RNA2	3588	
AF144234	South Korea	*Capsicum annuum*	<1999	Unknown	RNA1	5952	
AB018698	Japan	*Capsicum annuum*	<1998	IP	RNA2	3569	[76]
FN985164	China: Xinjiang	Tomato	2009	XJ14-3	RNA1	5955	
AF228423	China	*Chenopodium quinoa*	<2000	P158	RNA2	3597	
AB050782	Philippines	*Pogostemon cablin*	<1998	Philippines	RNA1	5956	
AB011007	Philippines	*Pogostemon cablin*	<1998	Philippines	RNA2	3591	

Table S2. Reassortment analysis by RDP software using 38 BBWV2 artificially concatenated sequences.

Isolate	Sequence Used to Infer Major Parent	Sequence Used to Infer Minor Parent	Recombination Detecting Program (*p*-value[a])						
			RDP	GENECONV	BOOTSCAN	MAXCHI	CHIMAERA	SISCAN	3SEQ
**AH**	**UN**[b]	BB9	1.315×10^{-165}	2.467×10^{-144}	3.920×10^{-153}	3.496×10^{-57}	1.422×10^{-9}	3.519×10^{-51}	2.202×10^{-12}
RP3	UN	SP	2.399×10^{-165}	7.723×10^{-144}	3.583×10^{-169}	1.109×10^{-49}	2.032×10^{-50}	3.640×10^{-52}	1.652×10^{-12}

P3	UN	SP	9.053×10^{-165}	6.756×10^{-144}	3.583×10^{-169}	1.109×10^{-49}	2.032×10^{-50}	3.640×10^{-52}	1.652×10^{-12}
P2	UN	SP	9.053×10^{-165}	6.756×10^{-144}	3.583×10^{-169}	1.109×10^{-49}	2.032×10^{-50}	3.640×10^{-52}	1.652×10^{-12}
BB5	UN	SP	9.053×10^{-165}	6.756×10^{-144}	3.583×10^{-169}	1.109×10^{-49}	2.032×10^{-50}	3.640×10^{-52}	1.652×10^{-12}
AB1	Ty	RP2	2.748×10^{-75}	1.510×10^{-77}	1.436×10^{-70}	1.195×10^{-45}	7.273×10^{-15}	1.274×10^{-92}	2.202×10^{-12}
ME	PAP1	LN	1.079×10^{-20}	3.434×10^{-12}	9.924×10^{-9}	3.165×10^{-28}	1.918×10^{-21}	3.932×10^{-9}	1.652×10^{-12}
RP7	UN	BB9	6.135×10^{-71}	7.695×10^{-73}	2.216×10^{-72}	1.040×10^{-35}	1.327×10^{-7}	2.845×10^{-89}	2.485×10^{-22}
BB2	BB9	SP	7.476×10^{-23}	8.905×10^{-11}	6.889×10^{-22}	6.192×10^{-16}	8.122×10^{-19}	5.264×10^{-23}	5.506×10^{-13}
IP	UN	BB9	6.135×10^{-71}	7.695×10^{-73}	2.216×10^{-72}	1.040×10^{-35}	1.327×10^{-7}	2.845×10^{-89}	2.485×10^{-22}

[a]The analyses were done using default settings and a Bonferroni-corrected *P*-values cut-off of 0.01 in RDP4 software. [b]UN, Unknown.
